# Characterization of Wing Kinematics by Decoupling Joint Movement in the Pigeon

**DOI:** 10.3390/biomimetics9090555

**Published:** 2024-09-15

**Authors:** Yishi Shen, Shi Zhang, Weimin Huang, Chengrui Shang, Tao Sun, Qing Shi

**Affiliations:** 1 Intelligent Robotics Institute, School of Mechatronical Engineering, Beijing Institute of Technology, Beijing 100081, China; sheny46cardiff@163.com (Y.S.); stone_2018@126.com (S.Z.); 13121119300@163.com (W.H.); shiqing@bit.edu.cn (Q.S.); 2 Key Laboratory of Biomimetic Robots and Systems, Beijing Institute of Technology, Ministry of Education, Beijing 100081, China; 3 Key Laboratory of Animal Ecology and Conservation Biology, Institute of Zoology, Chinese Academy of Sciences, Beijing 100101, China; shangchengrui21@ioz.ac.cn; 4 University of Chinese Academy of Sciences, Beijing 100049, China; 5 Yangtze Delta Region Academy of Beijing Institute of Technology, Jiaxing 314000, China

**Keywords:** flapping wing aerial vehicle, biomimetic robots, wing kinematics, mechanical design

## Abstract

Birds have remarkable flight capabilities due to their adaptive wing morphology. However, studying live birds is time-consuming and laborious, and obtaining information about the complete wingbeat cycle is difficult. To address this issue and provide a complete dataset, we recorded comprehensive motion capture wing trajectory data from five free-flying pigeons (Columba livia). Five key motion parameters are used to quantitatively characterize wing kinematics: flapping, sweeping, twisting, folding and bending. In addition, the forelimb skeleton is mapped using an open-chain three-bar mechanism model. By systematically evaluating the relationship of joint degrees of freedom (DOFs), we configured the model as a 3-DOF shoulder, 1-DOF elbow and 2-DOF wrist. Based on the correlation analysis between wingbeat kinematics and joint movement, we found that the strongly correlated shoulder and wrist roll within the stroke plane cause wing flap and bending. There is also a strong correlation between shoulder, elbow and wrist yaw out of the stroke plane, which causes wing sweep and fold. By simplifying the wing morphing, we developed three flapping wing robots, each with different DOFs inside and outside the stroke plane. This study provides insight into the design of flapping wing robots capable of mimicking the 3D wing motion of pigeons.

## 1. Introduction

Birds exhibit advanced flight capabilities, such as navigating through dense forests and executing evasive maneuvers, that are unmatched by existing fixed-wing [1] or rotary-wing robotic aircraft [2]. These abilities are enabled by a complex skeletal system that allows for wing morphing, rapidly and dramatically altering the magnitude and direction of aerodynamic forces [3,4]. Biomimetics has always impacted the real world. Through the study of remoras, researchers have developed robots that can cross-medium operations and can hitchhike [5]; Inspired by avian eyes, the perovskite artificial vision system to provide foveated and multispectral imaging was validated through imaging demonstrations [6]. Similarly, drones equipped with wing deformability and bird feathers have been shown to deform more quickly and robustly under aerodynamic loading [7]. Thus, understanding these wing morphing mechanisms can offer valuable insights for improving the flight performance of flapping vehicle designs.

Wing morphing refers to the physical ability of birds to dynamically morph their wings, enabling significant changes in lift and stability. Several key kinematics parameters can characterize this ability. However, previous studies have only briefly described the wing twist and camber of the house sparrow [8]. Another study on the wing surface reconstruction of the landing steppe eagle found that shoulder motion could be characterized by three parameters: pitch, dihedral, and sweep [9,10]. The complete wing shape during flapping was recently obtained from a barn owl in forward flight [11], and the motions (plunge, pitch, slide) and profile parameters of a single flight were assessed in detail. Some studies have focused on specific motion characteristics, such as the phase angle between pitching and flapping [12], wing bend motion [13], and wing fold motion [14], to investigate their effects on the aerodynamic performance of flapping wings. Despite recent progress, these studies are limited by selecting only a few kinematic parameters. Two main factors contribute to this constraint: First, the free flight of birds requires a large experimental field, which makes it difficult to arrange the camera to capture their movements. Second, the type and number of marker points utilized during the data acquisition process can potentially impact the acquisition of wingbeat data due to the proximity of two markers or obscuration by pigeon movement. For instance, excessive spherical markers can cause acquisition failures during flight. Together, these factors constrain the detailed description and analysis of wing kinematics in free-flying birds.

Even after quantifying wing kinematics, computing the forelimb joint movement angles is necessary to fully understand the functional kinematics of the forelimb joints in birds. A substantial number of previous studies have treated the bird’s forelimb as either a planar four-bar mechanism [15,16], a spatial six-bar mechanism containing two wrist bones [17,18], or a specific open kinematic chain starting at the shoulder where the radius and ulna fuse into a single bone [19,20]. These mechanisms provide information about joint types and angles. However, most data in previous studies have been derived from cadavers, which can differ from the actual flight dynamics of live birds, resulting in unrealistic joint postures. To address this, both anatomical structures and actual flight sequences should be considered when calculating joint angles. A previous study employed principal components analysis (PCA) to identify the simplest mechanism for replicating bird wing motion [17]. PCA decomposes the measurement data set into linear components, each representing a measurable portion of the total motion. This approach has previously been used to determine the complexity of bat wings [21] and to guide the design of a corresponding bat-inspired robot [22]. However, the results should be interpreted with caution, as the components are mathematical constructs in a high-dimensional space, and current high-performance actuators cannot fully replicate these components. Consequently, configuring DOFs and modeling real-time forelimb joint angle changes in live birds during flight remains a significant challenge.

To address these issues and enhance the understanding of flight mechanics and coupled control, the current study describes wing kinematics during continuous flapping of five free-flying pigeons ( Columba livia) while using the joint angles for the DOF configuration. To accurately capture the pigeons’ movements, we constructed a large flight arena (16 × 5 × 3 m) equipped with a motion capture system (MoCap; 30 cameras with infrared [IR] spectral sensitivity), and a monitoring system (MoTor; three GoPro cameras), which enabled tracking at 240 Hz with sub-millimeter accuracy. Based on this step, we obtained highly accurate three-dimensional (3D) positional and postural information of the pigeons’ wing anatomical points throughout their entire flight, from take-off to landing.

In this paper, we provide a large dataset of pigeon wing movements during leveling flight. To provide a comprehensive description of the wing motion, we divided the motion within a wingbeat cycle into five kinematic parameters: flapping ϕ, sweeping ψ, twisting θ, in-plane (stroke plane) bending Δϕ, and out-of-plane folding Δψ. Meanwhile, to obtain the pigeon’s real-time forelimb joint angle changes during flight, we built a three-bar open-chain kinematic model for model-to-skeleton mapping. Using a global optimization algorithm, we determined the shoulder, elbow, and wrist joint types and angles. Subsequently, we conducted a correlation analysis to explore the relationships between joint movements and the five key motions. Eventually, we found out that the shoulder and wrist roll angles were coupled during the upstroke, while the yaw angles of the shoulder, elbow, and wrist were coupled during both the upstroke and downstroke. Based on this, we developed three wing actuation mechanisms of pigeon-inspired robots whose wings can couple, morphing in and out of the stroke plane. In conclusion, the complex wing kinematics of pigeons have been analyzed, and joint movements have been revealed. It offers valuable insights for designing bird-inspired unmanned aerial vehicles (UAVs) with deformable and highly coupled wings.

The remainder of the article is structured as follows. In Section 2, the data collection process and flight arena are detailed, along with the proposed methodology for segmenting wing motions and the optimization process of the three-bar mechanism model. In Section 3, the analysis of wing kinematics and joint angles during the leveling flight phases is presented, along with their correlation and the three proposed wing actuation mechanisms. Section 4 provides an in-depth discussion of the results. Finally, in Section 5, the conclusions of this article are presented.

## 2. Materials and Methods

### 2.1. MoCap Setup and Experimental Pigeons

**Figure 1:**
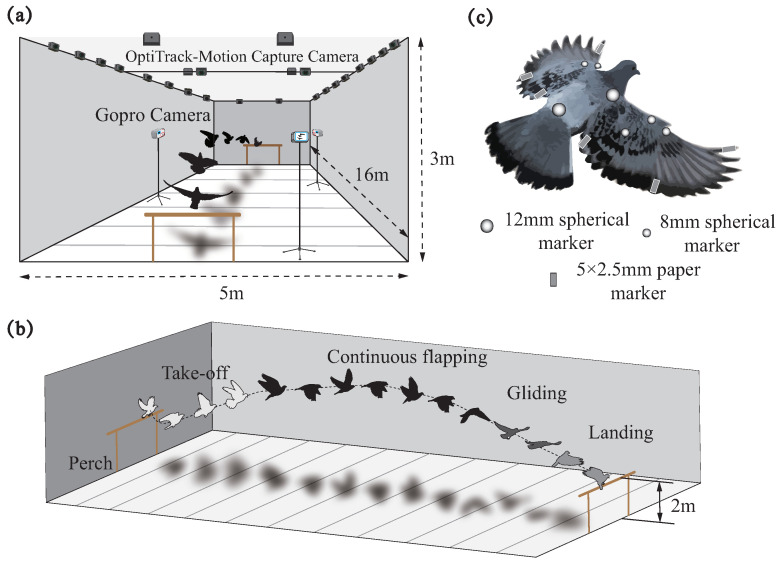
Schematic view of flight arena. (**a**) Overview of the measurement arena. The size of the experimental arena was 16 m × 5 m × 3 m, and the 30 motion capture cameras used were evenly distributed on the roof. At the same time, three GoPro cameras were also placed around the area to assist with the capture. (**b**) Regarding the four flight modes of pigeons during flight experiments, we only analyze the data for the continuous flapping phase in this paper. (**c**) The locations and names of the markers on the pigeons.

As shown in Figure 1a, experiments were recorded within a custom-built flight arena 16 m long × 5 m wide × 3 m high in size. Thirty motion-capture cameras (OptiTrack Prime 13) were used for recordings at 240 Hz with a resolution of 1280 × 1024 pixels and operated using OptiTrack Motive 2.3.4 Final software (NaturalPoint, Corvallis, OR, USA). All cameras were mounted on fixed scaffolding around the room at a distance of 3 m from the floor. Each calibration of the system was performed by hand using an OptiTrack CWM-250 calibration wand with a length of 250.018 ± 0.002 mm. The cameras were calibrated such that a marker within the recording volume would be visible to at least three cameras. The MoCap system tracked the visible markers in the recording volume and reconstructed their position into 3D coordinates with an accuracy of ∼0.5 mm. In addition, a monitoring system with three GoPro cameras (HERO9 Black) was used to assist with manual data processing (Figure 1a).

Experiments were performed with 2 to 3-year-old adult pigeons (*Columba livia*, hereafter ‘pigeons’). These pigeons were bred on-site from carrier pigeon stock, from which we selected five pigeons for all of the experiments. The pigeons’ morphological parameters are shown in Table S2. Approval of all ethical and experimental procedures and protocols was granted by the Experimental Animal Welfare Ethics Review Committee at the Institute of Zoology, Chinese Academy of Sciences. The experiments were considered not to pose any significant risk of causing pain, suffering, damage, or lasting harm to the animal involved. Birds were provided with food and water ad libitum.

After acclimatization to the experimental site, two plastic perching crates (50 cm long × 50 cm wide × 200 cm high) were placed within the scene, while a lightweight plastic rope was attached to the pigeon’s foot to prevent escape. The pigeons were trained regularly (∼2 h per day) with food rewards to fly between perches for several weeks. Eventually, four flight modes were achieved: take-off, continuous flapping, gliding, and landing, as shown in Figure 1b. During the experimental data capture, pigeons were placed on one side of the perch, a staff member started recording on the computer of the motion capture system, and then the pigeons took off autonomously and landed on the other side of the perch, which ended the capture.

### 2.2. μ CT Measurement and Markers Setup

**Figure 2:**
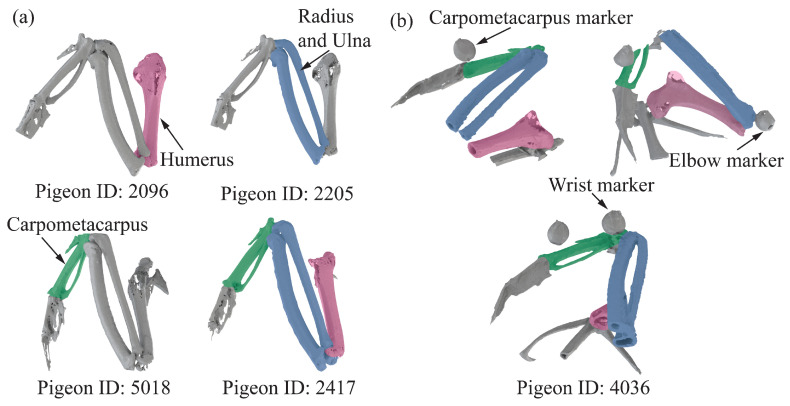
μ CT result forelimb skeleton 3D reconstruction for five pigeons. (**a**) Overall view of μ CT result for pigeon id: 2096, 2205, 5018, and 2417. It points out the humerus, radius, ulna, and carpometacarpus. (**b**) μ CT result for pigeon id 4036, the marker pasted on elbow, writs, and carpometacarpus.

μ CT provided a detailed 3D quantitative analysis of pigeon wing anatomy (e.g., bone length), guiding the placement of the markers. We scanned five pigeons after anesthesia using μ CT (PE Quantum FX), as shown in Figure S1. The pigeon’s wings are pulled back to ensure the μ CT can fully scan the forelimb skeleton. Then, we reconstructed the bones using 3D Slicer software Version 5.4.0 (www.slicer.org, accessed on 20 October 2023); the result can be seen in Figure 2a. We then measured the distance between every two joints in five pigeons and acquired the distance from shoulder to elbow, elbow to wrist, and the length of the carpometacarpus. Consider the distance from the shoulder to the elbow as the length of the humerus. Consider the distance from elbow to wrist as the length of the radius and ulna. The detailed results are shown in Table S1; each length of the forelimb skeleton was used to model open-chain kinematics later. Subsequently, three different sizes and styles of markers were pasted for different anatomical locations based on the measured skeleton lengths while considering the problem of markers’ self-obscuration during movement, as shown in Figure 1c and Movie S1. As shown in Figure 2b, to determine the offset distance between the marker points and the joints, we also measured the distance from the marker to the center of the joints to add these average offsets to the calculation in the subsequent optimization process.

Through hundreds of training and testing, we finally determined the scheme, which allowed for clear marker point observation. Specifically, three spherical markers with a diameter of 12 mm were attached to the body in the coronal plane: left shoulder, right shoulder, and rump. Three spherical markers with a diameter of 8 mm were attached to the leading edge of each wing: elbow, wrist, and carpometacarpus. Three rectangular tabbed markers 5 mm long and 2.5 mm wide were attached to the trailing edge of each wing: ninth primary, first secondary, and tenth secondary. All markers were removed after the experiment, leaving no visible damage to the feathers.

### 2.3. Three-Dimensional Position Reconstruction

After training, we recorded 15 flight trials per pigeon (Pigeon sample size *n* = 5) for 6 days, of which *n* = 6 quantitative samples of kinematics per species met quality control criteria for experimental analysis. Notably, the inbuilt labeling system of the OptiTrack Motive 2.3.4 is based on the assumption that the distance a marker moves between two frames is less than the distance between adjacent markers within a frame. In pigeon flight, on the one hand, the flight speed is high, and on the other hand, the feathers cover the marker when the wings are deformed; therefore, the OptiTrack system cannot consistently label each marker. We use the inbuilt manual labeling method to label the coordinates of the unlabeled markers.

Only 2.99% of the data are missing after manual filtering. Then, we used custom-written scripts, the raw position data were filled with missing data using cubic spline interpolation, followed by filtering using a fourth-order Butterworth filter with a low-pass cutoff frequency of five times the flapping frequency. To avoid poor performance of the Butterworth filter near the beginning and end of the time series, 15 frames of data at each end of each flight trial were omitted after filtering.

### 2.4. Coordinate Systems and Wing Movement

**Figure 3:**
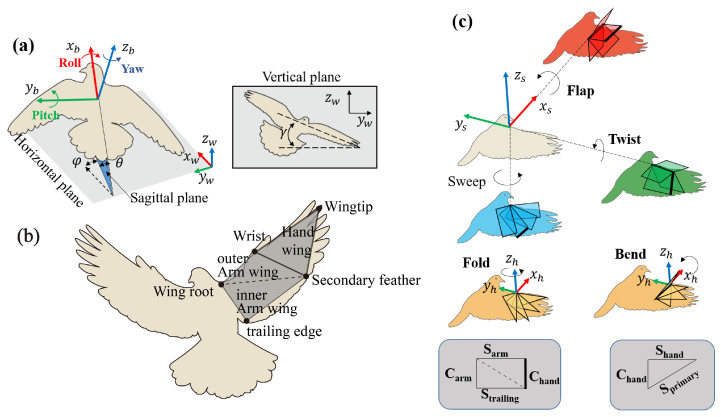
Definitions of the coordinate systems during flight. (**a**) Three Euler angles are used to describe the orientation of the pigeon’s body in the world coordinate system elevation: elevation ( Θ), heading ( Ψ), and bank angle ( Φ). The horizontal plane is shown in grey. (**b**) Recorded anatomical points on the wing (see Figure 1c) were used to define multiple planes. (**c**) Represent of the five angles in the arm wing and hand wing coordinate systems.

To describe the complex dynamic movement of the pigeon’s body, we introduce two right-handed coordinate systems as shown in Figure 3: the world coordinate system $O_{w}-x_{w}y_{w}z_{w}$ and the body-fixed coordinate system $O_{b}-x_{b}y_{b}z_{b}$. As shown in Figure 3a, $O_{w}-x_{w}y_{w}z_{w}$ is defined by the flight arena, $O_{b}$ fixed to rump, $x_{b}$ was described as a point forward and coincided with the line connecting rump with the midpoints of left shoulder and right shoulder, and the $y_{b}$ is connecting left shoulder and right shoulder and pointing to the left shoulder. Figure 3a shows the pigeon body orientation using a set of ordered Euler angles, i.e., elevation ( Θ), heading ( Ψ), and bank angle ( Φ). Rotations concerning the body-fixed system $x_{b}$, $y_{b}$, and $z_{b}$ are defined as roll, pitch, and yaw, respectively.

**Figure 4:**
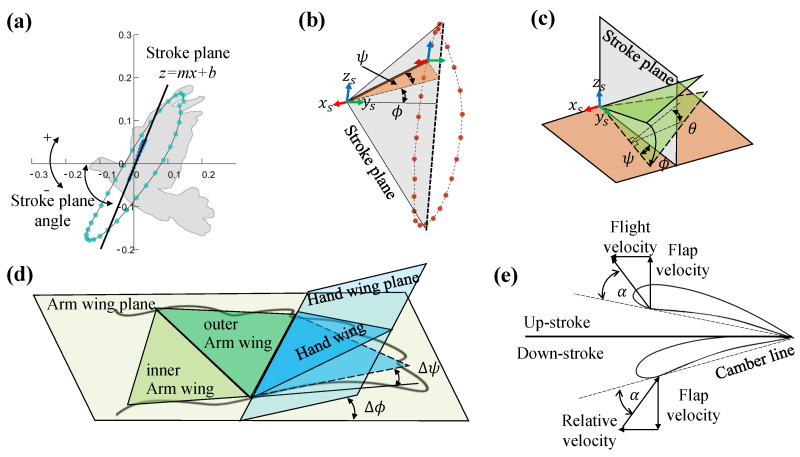
Definitions of the wing kinematics during continuous flapping. (**a**) The stroke plane corresponds to a linear regression plane of the *x* and *z* of the wrist joint relative to the shoulder. (**b**) The flap angle is between the wing plane and $x_{s}y_{s}$ plane. The sweep angle is between the leading edge and the stroke plane. (**c**) The twist angle is the wing chord length being rotated about the transverse $y_{s}$ axis. (**d**) The fold angle is the hand wing plane rotation along the $z_{h}$ axis. The bend angle is the hand wing plane rotation along the $x_{h}$ axis. (**e**) Schematic definition of wing angle of attack.

Figure 3b shows the wing kinematics during continuous flapping, we use the markers at key locations in the wing structure to simplify the pigeon wing into six planes: left inner arm wing, left outer arm wing, left hand wing, right inner arm wing, right outer arm wing, right hand wing. Here, we only focus on the right wing. Otherwise, two other coordinate systems are introduced: the arm wing coordinate system $O_{s}-x_{s}y_{s}z_{s}$ and the hand wing coordinate system $O_{h}-x_{h}y_{h}z_{h}$, as shown in Figure 3c. In Figure 4a, we obtained the stroke plane by performing a linear regression using the *x* and *z* coordinates of the wrist motion data relative to the shoulder. To eliminate the shoulder marker shaking, the origin $O_{s}$ of $O_{s}-x_{s}y_{s}z_{s}$ is the intersection of the stroke plane with the connecting line between the RU and the center of distribution of the shoulder in *x* and *z* coordinates. The $x_{s}$ is perpendicular to the xOz plane and points forward, the $z_{s}$ is in the stroke plane and points upward, the $y_{s}$ is orthogonal to the $x_{s}$ and $z_{s}$ and points to the left wing. The coordinate system of $O_{h}-x_{h}y_{h}z_{h}$ can be derived from the transform matrix of the coordinate system of $O_{s}-x_{s}y_{s}z_{s}$.

In Figure 3c, three motions in the $O_{s}-x_{s}y_{s}z_{s}$ coordinate system and two in the $O_{h}-x_{h}y_{h}z_{h}$ coordinate system were defined to express the 3D wing. The following movements are defined: flapping ( ϕ), sweeping ( ψ), twisting ( θ), in-plane(stroke plane) bending ( Δϕ), and out-of-plane folding ( Δψ). To reduce the complexity of the wing configurations, each two arm wing section is considered as a simple arm wing panel. Specifically, as demonstrated in Figure 4b,c, ϕ describes the rotation about the $x_{s}$, with the wingtip pointing (or tilting) dorsally if the flap angle was positive and ventrally if it was negative. ψ describes the angle of the wing rotation about the ventrodorsal $z_{s}$. A positive sweep indicates that the wing span is pointing forward, and at 0° the wing is perfectly perpendicular to the body. θ is the angle at which the wing chord length is rotated about the transverse $y_{s}$ axis, and a positive twist angle will bring the trailing edge down and forward. The three kinematics described above are used to describe the motion of the arm wing plane relative to the body frame. In the hand wing, Δϕ is aligned with ϕ to describe rotation about the $x_{h}$, with the wingtip pointing dorsally when the bend angle is positive, and ventrally when the bend angle is negative. Finally, Δψ describes the rotation about the $z_{h}$, and the positive fold indicates that the wing span is pointing forward.

As illustrated in Figure 4e, the angle of attack (AOA) of the arm wing and the hand wing is calculated based on the position of the chord of the arm wing plane and the hand wing plane relative to the airflow vectors through the chord (the vector sum of the wing flap speed and the forward speed).

We pre-parameterized the above motions with the Fourier series to compare the wing kinematics between individuals. The sweep angle has a small range of variation and is described by a second-order Fourier series, while a first-order Fourier series describes the other four angles:

(1) $$\phi \left(\hat{t}\right)=\phi_{0}+\phi_{s}sin\left(2\pi \hat{t}\right)+\phi_{c}cos\left(2\pi \hat{t}\right),$$

(2) $$\psi \left(\hat{t}\right)=\psi_{0}+\sum_{i=1}^{2}\psi_{si}sin\left(2\pi i\hat{t}\right)+\sum_{i=1}^{2}\psi_{ci}cos\left(2\pi i\hat{t}\right),$$

(3) $$\theta \left(\hat{t}\right)=\theta_{0}+\theta_{si}sin\left(2\pi \hat{t}\right)+\theta_{ci}cos\left(2\pi \hat{t}\right),$$

(4) $$\Delta \phi \left(\hat{t}\right)=\Delta \phi_{0}+\Delta \phi_{s}sin\left(2\pi \hat{t}\right)+\Delta \phi_{c}cos\left(2\pi \hat{t}\right),$$

(5) $$\Delta \psi \left(\hat{t}\right)=\Delta \psi_{0}+\Delta \psi_{s}sin\left(2\pi \hat{t}\right)+\Delta \psi_{c}cos\left(2\pi \hat{t}\right),$$

where $\hat{t}$ is the dimensionless time (change from 0 to 1 in one wingbeat cycle), $\phi_{0}$, $\psi_{0}$ and $\theta_{0}$, etc., are the coefficients of the harmonics chosen to give the best least squares fit to the wing kinematic angles.

### 2.5. Pigeon Forelimb Kinematic Model and Mapping Process

**Figure 5:**
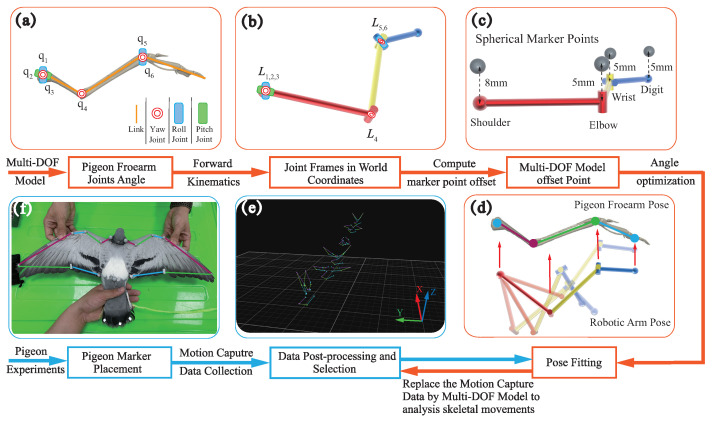
Schematic diagram of the mapping process using the proposed hierarchical global optimization algorithm for computing joint angles. The framework consists of two layers. The upper layer (red box) built a three-bar mechanism based on an open chain characterizing the pigeon forelimb skeleton. The lower layer (blue box) mainly concerns flight data acquisition and forward kinematics iteration. (**a**) The DOF of the joint angle is determined. (**b**) The OKC model in the world coordinates. (**c**) The offset of the marker points on each joint concerning the OKC model. (**d**) The optimization process is to fit the corrected OKC model pose to the capture position pose and the output of the joint angles. (**e**) Capture data visualization and pre-processing in a motion capture system. (**f**) The marker placement on the pigeon.

A hierarchical optimization algorithm is employed to map the model to the forelimb, thereby facilitating the calculation of the angle of the joints during wingbeat, the process is shown in Figure 5. The algorithmic framework includes two main layers: (1) in the upper layer, a three-bar mechanism based on an open kinematic chain (OKC) is constructed to represent the pigeon forelimb skeletal system, and simultaneously the offset values of the markers relative to the joints in the model are determined with the assistance of μ CT; (2) in the lower layer, the positional data of the pigeon’s shoulder, elbow, wrist, and carpometacarpus are recorded using the MoCap, and finally, the joint angles are obtained by minimizing the Euclidean distance of the corresponding markers between the mechanism and the flight data. Each layer of the algorithmic framework is described in detail below.

Given the complex skeletal system of the pigeon forelimb, a quantitative model that omits some anatomical features is needed to describe the joint motions. This study used a simplified three-bar mechanism to analyze joint motion. At the same time, more realistic anatomical features were contemplated to characterize the type of joints. Specifically, as shown in Figure S2, we merged the radius and ulna to obtain a mechanism containing three parts: hand, forearm, and arm.

In addition, because of the restricted motion of the elbow, we considered it as a pin joint with one DOF (pitch), and the other two joints, the shoulder and wrist, as spherical joints with three DOFs (pitch, yaw, and roll). Thus, the state of the three-bar mechanism is described as follows:

(6) $$x=[\begin{array}{cccccc} q_{s}^{yaw} & q_{s}^{roll} & q_{s}^{pitch} & q_{e}^{yaw} & q_{w}^{yaw} & q_{w}^{roll} \end{array}]$$

*q* denotes the angle of the joint, and the subscript indicates the type of joint, *s*, *e*, and *w* for shoulder, elbow, and wrist. Note, that the exact joint types are described in Section 3.3.

The next task was to identify the joint angles, denoted by x, to obtain the coordinates of each joint so that the three-bar mechanism closely matches the pigeon forearms during the corresponding flight movement. We used a modified Denavit–Hartenberg (DH) method to perform forward kinematics. However, one challenge was that the markers were attached to the pigeon’s feathers and did not truly reflect the position of the joints. As shown in Figure 5c, the position offset of markers relative to the elbow, wrist, and carpometacarpus was determined to be 0.005 m by μ CT. The relationship between the positions of these markers and the joint positions is as follows:

(7) Pw=Tmw×000.0051

where Tmw denotes the homogeneous matrix of the frame in which markers are located to the world frame, and Pw is the position of markers in the world frame. More specifically, the shoulder joint has an offset of 0.008 m, but it is not involved in the optimization process. It just moves the *z*-axis of the three-bar mechanism along the negative direction by 0.008 m to align it with the origin of the flight data due to the length of the holder for placing the marker points being 0.008 m.

For the second layer of the optimization algorithm, we obtained flight position data for the pigeon containing the shoulder, elbow, wrist, and carpometacarpus in Section 2.3. After obtaining two comparable point sets, the mapping problem became finding the appropriate joint angle **x** such that the markers of the three-bar mechanism match those of the flying pigeon. Accordingly, we defined the objective function f(x) as the minimum Euclidean distance of the corresponding markers between the collected joint and the established mechanism. Furthermore, considering that the three joints of the model resulted in different contributions to the minimization objective function, we added three weighting factors to adjust it, using the following equation:

$$min\;f\left(x\right)=\frac{\sum_{i=1}^{3}c_{i}*dist{\left(P_{tracked}^{model}\right)}_{i}}{3}$$

(8) $$\begin{array}{cc} \; & \;\;\;s.t.\left\{\begin{array}{c} c_{i}\left(i=1,2,3\right)>0\\ x_{low}\leq x\leq x_{up} \end{array}\right.\\ \; & \;\;x_{low}=\left[-\frac{2}{3}\pi ,-\frac{1}{3}\pi ,0,-\frac{3}{5}\pi ,\frac{1}{2}\pi ,-\frac{1}{2}\pi \right];\\ \; & \;\;x_{up}\;\;=\left[\;0\;,\;\frac{1}{3}\pi ,\frac{1}{4}\pi ,\pi ,\;\;\frac{4}{3}\pi ,\;\;\;\frac{1}{6}\pi \right]; \end{array}$$

where $\;c_{1}$, $\;c_{2}$, and $\;c_{3}$ are the weight coefficients and satisfy $\;c_{1}\in \left(0,1\right)$, $\;c_{2}\in \left(0,1\right)$ and $\;c_{3}\in \left(0,1\right)$. The choice of weight coefficients during optimization is based on the DOFs corresponding to each joint. We took the 3 DOFs shoulder as $\;c_{1}=0.60$, 1 DOF elbow as $\;c_{2}=0.15$ and 2 DOFs wrist as $c_{3}=0.15$. The constraints and initial values were crucial in the above nonlinear optimization problem. Because the optimization process is coupled, if the constraints on a particular joint angle are too tight, the angle may reach a position the actual bone cannot reach to meet the optimization objective, resulting in inaccurate joint angles. We defined the upper and lower bounds of the joints regarding studies on avian joints [17,23], as shown in Equation (8).

When dealing with the initial value problem, MoCap data were processed frame-by-frame, meaning that the position and orientation of the pigeon’s body did not change significantly from one frame to the next. Using the results of the previous frame as an initial guess for the current frame mapping provided an appropriate strategy to solve the problem. As an exception, the first frame was related to the flapping and bending angles calculated in Section 2.3. This accelerated convergence and improved the quality of the solution.

With the above description, the optimization algorithm is fully defined. To find a numerical solution to this problem, we used MATLAB fmincon library. The optimization is conducted when the objective function falls in the feasible direction and the final solution is within the optimal tolerance. However, other hyperparameters can be fine-tuned to improve the performance of the optimization algorithm.

As shown in Table S3, these parameters are carefully chosen through iterations to ensure the robustness and accuracy of the mapping algorithm. We can directly obtain the specific angle changes for each joint through optimization, enabling us to study the mechanics during pigeon free-flight better.

Time was normalized by the wingbeat period and then linearly interpolated for each variable, generating 50 time series data points per wingbeat. Each pigeon ID’s mean and s.d. are expressed as mean ± s.d. We also performed a one-way ANOVA using MATLAB (MathWorks Inc., Natick, MA, USA) to determine if the pigeons had a statistically significant difference in kinematics. A *p*-value of greater than 0.05 was considered not statistically significant.

## 3. Results

### 3.1. Elucidate Wing Kinematics in Continuous Flapping Phase

**Figure 6:**
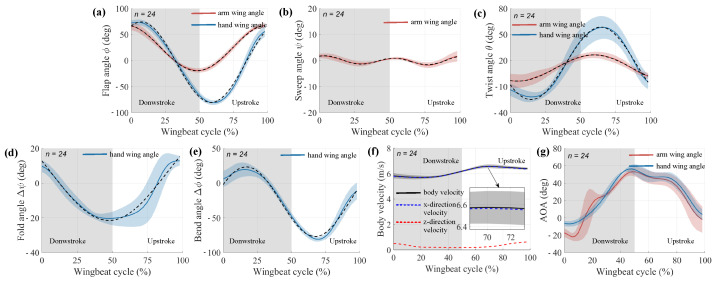
Averaged wing kinematics of pigeon ID 4036 in a normalized wingbeat cycle during continuous flapping. The solid line represents the mean traces, the shaded area indicates ±1 s.d. (*n* = 24), and the dashed line is the curve fitted to the Fourier series. Colors are used to represent different wing positions: red for the wrist and blue for the ninth primary. The white and grey backgrounds represent upstroke and downstroke, respectively. (**a**–**e**) flap angle ( ϕ), sweep angle ( ψ), twist angle ( θ), in-plane bend angle ( Δϕ), and out-of plane fold angle ( Δψ) in a normalized wingbeat cycle, respectively. (**f**) Pigeon body velocities, the solid black line shows the sum of the velocities, the dashed blue line shows in the x-direction and the dashed red line shows in the z-direction. (**g**) Angle of attack for arm wing and hand wing.

The wing kinematics during continuous flapping of pigeon ID 4036 in both the arm wing and hand wing planes are summarized in Figure 6. Mean values are shown for the arm-wing plane (red) and the hand-wing plane (blue), and the shaded area around the curve indicates s.d. (*n* = 24). In addition, to characterize intra-individual differences in kinematics, we fitted the above five kinematics with the Fourier series and extracted the magnitude and period of the fitted function as a comparison metric. Average wingbeat periods of different pigeons were 0.120 ± 0.005 s, 0.123 ± 0.010 s, 0.122 ± 0.011 s, 0.127 ± 0.012 s, and 0.132 ± 0.005 s, and did not differ significantly between individuals (*p* = 0.3156).

The wing kinematics ( ϕ, ψ, θ) derived from the proximal plane relates to the range of motion of the shoulder joint. The durations of downstroke and upstroke were almost identical. ϕ decreased from 65.25 ± 5.37 deg to −17.33 ± 5.3 deg in the downstroke, then increased to 70.58 ± 7.3 deg. The mean ϕ over a wingbeat cycle was 21.82 ± 2.93 deg.

In contrast to the traditional design idea of a flapping wing robot, which uses a symmetrical cosine curve as the wingbeat curve, pigeons have a large upstroke angle (red curve in Figure 6a). This cosine trend can also be observed in the hand-wing plane, where it decreases from 67.94 ± 4.57 deg to −81.24 ± 5.46 deg on the downstroke, then increases back to 52.82 ± 6.09 deg, with an average angle of −8.36 ± 2.8 deg in a wingbeat cycle. There are only two differences: first, the presence of a phase delay of 0.1 T along the spanwise position was observed, and second, the downstroke amplitude increased. The presence of in-plane Δϕ was responsible for this phenomenon. The sweep angle ψ has a small amplitude range (−2.9 deg to 3.6 deg).

Pigeons twist their wings during both downstroke (negative values) and upstroke (positive values), with a greater angle for upstroke. In the downstroke, there is a tendency for θ to decrease from proximal to distal (i.e., “washout”). As a result, the distal leading edge points more downward than the more proximal plane. On the upstroke, the sign is reversed and there is a large spreading twist in the distal plane. As torsion reverses, the ventral side of the wing faces upward, so the leading edge of the distal plane is more downward than the leading edge of the proximal plane, which again exhibits washout. This wing twist pattern is similar to the washout design used for propellers and helicopter blades, which is implemented to reduce induced power losses in the wake, thereby improving aerodynamic efficiency.

The wing kinematics derived from the hand wing plane have been referred to as “wing morphing” in some studies. However, considering that the forelimb skeletal muscle system can provide more DOFs, the “motion” was still used in this study to describe the movement of the hand wing. The in-plane Δϕ exhibited a positive (upward bend) value in downstroke (max. 19.82 ± 5.1 deg) and a negative (downward bend) value in upstroke (max. −82.1 ± 6.7 deg). The out-of-stroke Δψ is essentially non-positive in the upstroke and downstrokes (min. 25.03 ± 9.3 deg). Thus, in contrast to Δϕ (upward and downward), Δψ occurs mainly in the backward direction (Figure 6d,e).

The forward body velocity of the pigeon was 6.13 m/s during one wingbeat cycle, while we observed that the velocity during the downstroke was less than that during the upstroke. In addition, the velocity of the pigeon fluctuated very little in the vertical direction (*z*-axis), which means that the effect of drag was less during the upstroke of the pigeon. We also observed less difference in the angle of attack between the hand wing and the arm wing.

The *p*-values shown in Figure S4 for the magnitudes of flap, sweep, twist, bend, and fold angles were 0.2910, 0.1552, 0.1078, 0.0782, and 0.4439, respectively, indicating that they were not significantly different between individuals.

### 3.2. Systematic Evaluation of Joint Configurations and Qualitative Analysis of Angles

Owing to the interdependence of joint angles, determining the precise configurations of joint types requires cluster analysis and error comparison. For biological plausibility, the elbow joint had a single DOF, while the wrist and shoulder joints had three DOFs giving a total of 49 potential configurations. Throughout this investigation, we systematically constrained one or more angles, effectively reducing the DOF across the joints, and subsequently examined the average error across the three joints. Figure S5a comprehensively depicts all conceivable combinations, with the accompanying bar chart delineating the associated errors for each combination. Based on the analysis of the error, the three joints are represented as a spherical joint for the shoulder joint (3-DOFs), a pin joint for the elbow joint (1-DOF), and a universal joint for the wrist joint (2-DOFs). (electronic Supplementary Material, Figure S5).

**Figure 7:**
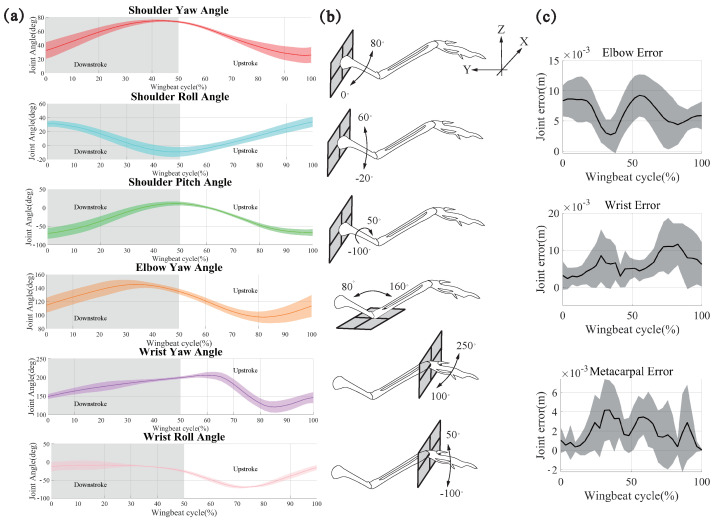
Joint movements and joint error of pigeon ID 4036 during continuous flapping. (**a**) The joint angles within one flapping cycle are illustrated for the joint DOF configuration of 3-1-2; they represent, respectively, shoulder yaw angle, shoulder roll angle, shoulder pitch angle, elbow yaw angle, wrist yaw angle, and wrist roll angle. The color bands represent each angle’s maximum and minimum values, and the colored solid lines indicate the average values. (**b**) Schematic representations of the magnitude and direction of the change in each joint angle. (**c**) Compared to the collected data, the optimized errors for the shoulder, wrist, and carpometacarpus.

Figure 7 shows the joint movements of pigeon ID 4036. The movements were defined using the shoulder frame. For simplicity, we used unified terminology to describe the above movements (Figure 7b): yaw motion implies protraction-retraction of the humerus, flexion-extension of the elbow, or adduction-abduction of the wrist; roll motion implies depression and elevation of the shoulder or wrist; pitch motion implies long-axis rotation of the humerus. Similar to the description of wing kinematics, we used the roll motion of the humerus to divide the wingbeat cycle into two phases: downstroke and upstroke.

As shown in Figure 7a, due to the joint angle constraints on the model, the yaw angle of the shoulder joint gradually increases during a wingbeat cycle on the downstroke. Then, it decreases gradually on the upstroke, which proves that there is an obvious forward sweeping movement of the bone on the downstroke, and the amplitude of this angle reaches about 50 degs. The results show that in the up and down strokes, the angle changes are symmetrical, so the shoulder joint’s sweeping is a regular reciprocating motion during the continuous flapping. The Yaw angle decreases slightly and then increases on the downstroke and the reciprocal motion corresponding to the up and down strokes. This change in angle causes the humerus to reciprocate in the −z to z direction, and this motion is the up-and-down flap of the pigeon, which is the predominant mode of motion of pigeons during flight. The roll angle of the shoulder joint has a wide range of variations. The roll of the humerus skeleton is based on the change in the yaw and pitch angles during the pigeon’s movement. The change in the roll angle makes the pigeon’s wings twist during flapping, and at the same time, it flexibly changes the wings’ AOA, improving flight performance. Figure S6 shows no significant variability in the angle of change in the shoulder joint between the five pigeons, which were all extremely similar (*p* = 0.672, 0.522, 0.578).

For the elbow joint, there is only one DOF. The amplitude of this angle varies between the extension of the Radius and Ulna in the Y-positive direction on the upstroke. The contraction is in the −Y direction on the downstroke and this movement causes the wing to contract in the XY plane, that is to say, the contraction of the wing due to the change in the elbow joints in the course of pigeon’s flapping, and the regular symmetrical variations in the upstroke and the downstroke prove that the elbow joint changes are closely related to the up and down strokes. Figure S6 shows that the five pigeons are similar up to *p* = 0.925, and there is no significant difference in the variation process.

The two DOFs of the metacarpal joint tend to have a greater influence on the deformation of the wing, which is to roll and yaw around the metacarpal joint. The yaw of the metacarpal joint varies more gently and incrementally on the downstroke during the wingbeat cycle, i.e., the metacarpus is folded in the X-negative direction, and more so on the upstroke, where it is folded back and forward, then back to its original position again. The metacarpus first folds back and forward then folds again in the X-negative direction and returns to its original position. During a wingbeat cycle, the pigeon changes the wing by constantly changing the folding and contraction of the metacarpus in the xy plane. The change in roll angle was more symmetrical, with the metacarpus bending slightly in the positive direction of the *z*-axis on the downstroke. However, this amplitude was not large and was followed by a gradual downward bending and reached a maximum at 3/5 of the way through the upstroke, followed by a gradual upward bending; this change in the degree of freedom caused a bend in the carpal joint. Moreover, the variability between the five pigeons was *p* = 0.056 and *p* = 0061, respectively; the five pigeons were also not significantly different.

### 3.3. Analysis of the Coupling between Wing Kinematics and Joint Movement

In this section, we focused on the couple relationship between joint movements and wing kinematics. Because both joint movements and five wing kinematics parameters were time-series vectors, the Pearson correlation coefficient ρ was used to illustrate the similarity between them:

(9) $$\rho \left(u_{wing},u_{joint}\right)=\frac{cov(u_{wing},u_{joint})}{\sigma_{wing}\sigma_{joint}}$$

**Figure 8:**
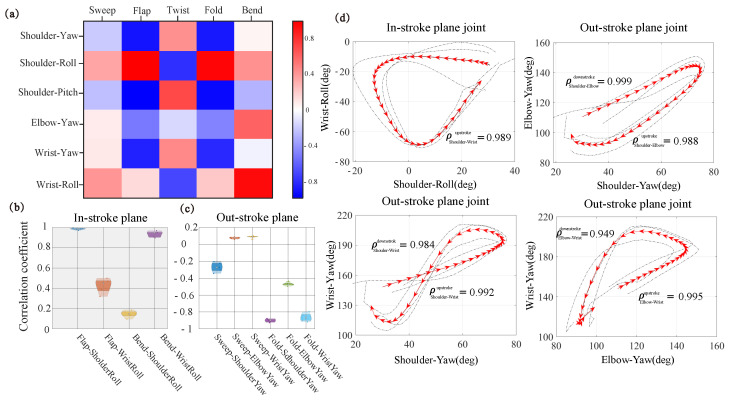
Wing kinematics and joint movements correlation analysis during continuous flapping. The analysis is based on a sample size of *N* = 5. (**a**) The color scheme depicts the correlation between each joint movement and wing kinematics, with red indicating a highly positive correlation and blue indicating a highly negative correlation. (**b**) The specific ρ between the two joint movements and two wing kinematics in and out of the stroke plane. (**c**) The specific ρ between the three joint movements and two wing kinematics out-stroke plane. (**d**) The correlation between wrist roll and shoulder roll, with arrows indicating the trend from the beginning of the downstroke to the end of the upstroke. In the upstroke, the correlation coefficient is $\rho_{Shoulder-Wrist}^{upstroke}=0.989$. The correlation between elbow yaw and wrist yaw of downstroke is $\rho_{Shoulder-Elbow}^{downstroke}=0.999$, and during the upstroke is $\rho_{Shoulder-Elbow}^{upstroke}=0.988$. The correlation between shoulder wrist yaw is $\rho_{Shoulder-Wrist}^{downstroke}=0.984$, and the correlation coefficient of upstroke is $\rho_{Shoulder-Wrist}^{upstroke}=0.992$. The correlation between elbow wrist yaw during the downstroke is $\rho_{Elbow-Wrist}^{downstroke}=0.995$, and the correlation of upstroke is $\rho_{Elbow-Wrist}^{upstroke}=0.949$.

Wing kinematics within the stroke plane included wing flapping and wing bending, as illustrated in Figure 4. Similarly, joint movement within the stroke plane involves rolls at the shoulder and wrist. Therefore, Figure 8b shows the correlation coefficients ρ between these two wing kinematics and joint movements. The strong correlation ( ρ=0.982308) between shoulder roll and wing flap indicates that the shoulder’s roll angle directly influenced the wing’s flapping. However, a strong correlation existed between wing bend and shoulder yaw ρ=0.388782 and wrist yaw ρ=0.93056. This result indicates that the variation in the bend angle was directly influenced by the wrist roll and exhibited a similar motion trend to the shoulder roll within the wingbeat cycle.

The wing kinematics outside the stroke plane included wing twist, sweep, and fold, as shown in Figure 8c. Joint motion in this context involved shoulder pitch, shoulder yaw, elbow yaw, and wrist yaw. Wing twist only strongly correlated with shoulder pitch, with a correlation coefficient of ρ=0.713746. The shoulder yaw most strongly influenced wing sweep, with correlation coefficients reaching ρ=−0.20402. In contrast, unlike sweep, the fold was primarily influenced by shoulder yaw, elbow yaw, and wrist yaw, with a correlation coefficient of ρ=−0.89743 for the shoulder joint, ρ=−0.47581 for the elbow joint and ρ=−0.83643 for the wrist joint.

The correlation analysis inside and outside the stroke plane shows that the joint movements were strongly correlated with wingbeat movements. As shown in Figure 8d, the direction of the arrow represents the beginning of the downstroke to the end of the upstroke. Within the stroke plane, there was a coupling relationship between shoulder and wrist rolls. During the downstroke, there was no clear correlation between shoulder roll and wrist roll, demonstrating that they did not exhibit the same trend in this phase.

However, in the alternating process of up and down strokes, the correlation between these two angles indicated that the changes in both angles were opposite, causing a phase lag; when the shoulder joint was already moving upward, the wrist joint was still moving downward. Subsequently, in the later stages of the upstroke, the wrist joint moved upward, similar to the shoulder joint ( $\rho_{Shoulder-Wrist}^{upstroke}=0.989$), and maintained a consistent motion trend. Thus, the wrist roll mainly affected the wing’s bending.

Outside the stroke plane, shoulder yaw and elbow yaw exhibited a strong correlation during both downstroke( $\rho_{Shoulder-Elbow}^{downstroke}=0.999$) and upstroke ( $\rho_{Shoulder-Elbow}^{upstroke}=0.988$). Additionally, elbow yaw and wrist yaw also showed strong coupled motion with both strokes( $\rho_{Elbow-Wrist}^{downstroke}=0.995$ and $\rho_{Elbow-Wrist}^{upstroke}=0.949$). At the same time, the yaw of the shoulder joint was strongly correlated with the yaw of the wrist joint ( $\rho_{Shoulder-Wrist}^{upstroke}=0.992$ and $\rho_{Shoulder-Wrist}^{downstroke}=0.984$). This demonstrated that, during the downstroke, when the shoulder joint swept forward, the elbow joint extended along the direction of the wing span, and simultaneously, the wrist joint extended. During the upstroke, when the shoulder swept back, the elbow joint flexed along the spanwise direction, and the wrist joint flexed accordingly. Therefore, the tendency of movement was the same for all three joints.

In addition, Figure 8c showed a large correlation coefficient between the sweep and yaw of the shoulder joint and a small correlation coefficient between the sweep and yaw of the elbow and wrist joints, because the simultaneous extension and flexion of the elbow and wrist joints resulted in a phase shift of the wrist joint concerning the shoulder joint. Thus, the sweep of the wing was mainly affected by the yaw of the shoulder joint.

### 3.4. Pigeon Inspired Robot

Robotics is frequently employed as a crucial methodology for validating biological mechanisms. Consequently, the utilization of robots with deformable wings became a pivotal aspect of the investigation to examine the superior flight performance of pigeons. In previous sections, we have summarized pigeons’ skeletal movement and wing kinematics as coupled motions of height correlation in and out of the stroke plane. Based on this summary, we have proposed three coupling approaches to achieve coupled motions in and out of the stroke plane of an inspired robot.

**Figure 9:**
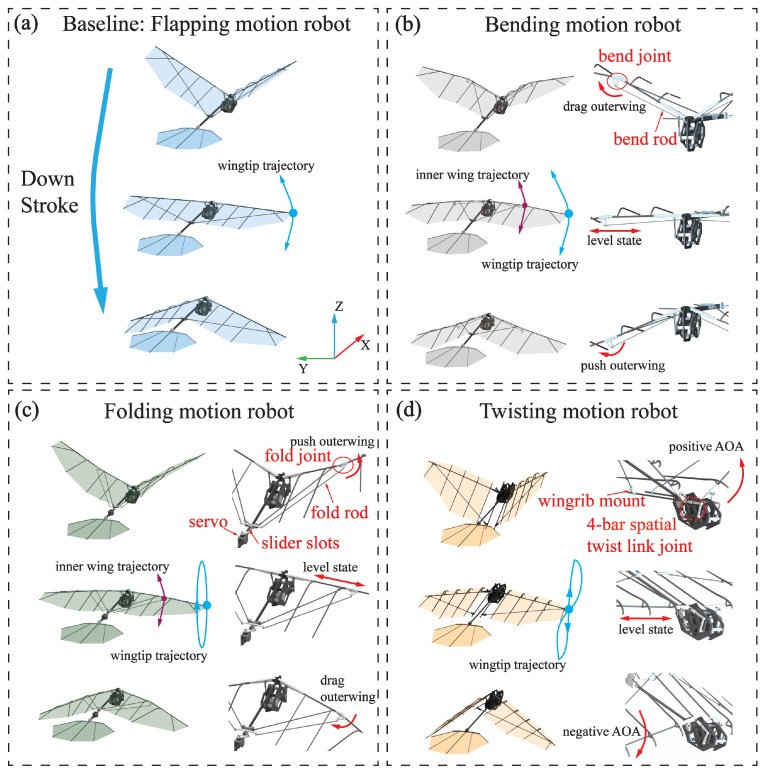
Pigeon-inspired robots with four different motions. (**a**) Flapping motion robot with only one DOF of the wing. (**b**) Bending motion robot, the inner and outer wings have different trajectories, both are in-stroke planes. The bend joint changes depending on the state of motion. (**c**) Folding motion robot, the folding of the outer wings is driven by the servo at the tail. (**d**) The twisting motion robot, twisting out of the stroke plane is achieved by an additional 4-bar spatial link to change the AOA of the wing.

The motion in-stroke plane is illustrated in Figure 9a,b. In Figure 9a, the pigeon-inspired robot exhibits a single DOF in-stroke plane, sufficient for the flapping motion. The driving mechanism requires only a spatial four-bar mechanism to drive the wings to flap. This single DOF is the most crucial and fundamental motion form in flight. Figure 9b illustrates the utilization of a rotating sub-joint at the wrist joint, analogous to that observed in the pigeon. This is connected to the rotating sub-joint by a bend rod. Additionally, the other end of the rod is linked to the flap crank at the root of the wing. This enables the bending motion at the wrist joint to be achieved by pulling back the linkage on the upstroke. This results in an inward constriction of the outer wing and a pushing out of the linkage on the downstroke, which leads to an outward expansion of the outer wing. This motion has been demonstrated to minimize vertical acceleration and maintain fuselage stability while increasing the longitudinal stability of the wings [24]. Because it effectively reduces the projected area during the upstroke, more lift can be obtained compared to the flap motion.

The motion out-stroke plane is illustrated in Figure 9c,d. As illustrated in Figure 9c, the slider moves back and forth in the sliding slot driven by the servo, while the folding joint at the wrist joint is fixed with the slider by the folding rod. During the upstroke, the rudder can be pushed outward by pushing the slider, resulting in outward folding of the outer wing. Conversely, during the downstroke, the rudder can be pulled backward by pulling the slider, allowing the outer wing to be folded inward, thereby achieving the wing folding motion. Consequently, this motion can obtain higher speeds in low-Reynolds-number and low-frequency flapping flight while enhancing flight stability. The pigeon-inspired robot depicted in Figure 9d has a distinctive torsion space four-bar mechanism that enables its wings to attain a torsion out-stroke plane. This allows for a substantial AOA during the upstroke, which is then modified to a smaller AOA during the downstroke. This oscillatory motion alters the AOA of the wing motion, thereby augmenting the lift generated during flight.

The design of the pigeon-inspired robot in and out of the stroke plane, using the coupled motion of the pigeon in different conditions can better guide the design of the flapping wing robot. Furthermore, the objective is to propose an innovative design concept for flapping-wing aircraft.

## 4. Discussion

This study aims to quantitatively analyze the wing kinematics and joint movement of pigeons during free flight, we captured five pigeons during free flight and quantified the wing kinematics as five parameters inside and outside of the stroke plane: flapping, sweeping, twisting, bending, and folding. Meanwhile, an open-chain kinematic model of the pigeon’s wing was developed. By systematic evaluation of the joint DOF configurations, we determined the DOF of each joint in the model: 3-DOF shoulders, 1-DOF elbows, and 2-DOF wrists. We also analyzed the detailed coupling between wing kinematics and joint movement in and out of the stroke plane. Based on this analysis, we propose three straightforward methods for designing flapping wing robots to achieve wing bending in the stroke plane and folding and twisting out of the stroke plane.

Following extensive training of pigeons and using reflective marker paste, 30 motion capture cameras were employed to document the essential characteristics of the pigeons’ wings during free flight. The acquisition process is more comprehensive, thereby ensuring that the pigeons can accurately perform the process of continuous flight during the experiment and guaranteeing the data’s reliability. Different from the collection process of small birds or insects [25,26], pigeons are more uncontrollable yet frequently employed as bionic objects due to their superior flight abilities over long and short distances [27]. The extensive flight data yielded by our research can serve as a valuable source of information for designing bionic mechanisms.

The present study analyses the wing morphing of living pigeons in conjunction with joint movements, filling a gap in the existing literature for medium-sized birds. To represent the wing deformation more comprehensively, the wing was divided into three surfaces. This approach allows for a comprehensive description of the deformation of the wing during the wingbeat cycle. The same methodology was employed for the analysis of the wing angle [28]. Additionally, this study presents, for the first time, the angular transformation of the wing forelimb skeleton during free flight. Furthermore, it differs from previous studies in that it does not utilize bird carcasses as the object of skeletal transformation studies [18]. Our methodology is based on the DH method, which allows us to map our open-chain motion model to free-flight joint movements to obtain the pigeon’s real-time angular changes during flight. In addition, the wing morphing resulting from the angular changes in each joint during the up and down strokes is subjected to comprehensive analysis. By analyzing the correlation between the various moments of the up and down strokes, it can be derived that it is not a specific joint movement that directly causes the deformation of the wing. Furthermore, it can be observed that these mutually coupled effects differ during the up and down strokes. In contrast to engineering problems, as natural flyers, pigeons possess an inherent advantage in controlling flight by altering their wing shape. Consequently, understanding the specific movement the skeleton makes at specific moments to affect the deformation of the wings is of significant value for the development of bionic flapping-wing vehicles or mechanisms with deformable wings [29,30]. This knowledge can inform the design of a more rational bionic mechanism and guide the actuation and control methods employed. The findings inform the design of more rational bionic mechanisms and guide the optimal drive and control methods.

This paper also presents three flapping wing robots with different bionic deformation structures. These robots achieve wing deformation in and out of the stroke plane through a single actuation method or a clever coupling design. Furthermore, all of these robots have the same dimensions as pigeons. While it has been demonstrated that these mechanisms function similarly to pigeons in terms of morphology, their exact flight performance is still unknown. The aerodynamic aspects of this paper have been addressed only in a limited manner, and thus, the aerodynamic and flight performance of the flapping wing prototypes require further investigation.

In future work, it would be beneficial to analyze the wing kinematic transformations in greater detail during the take-off and landing phases. During the smooth flight phase, pigeon wings beat with stability; however, pigeons require a change in their flight state during both the take-off and landing phases. Consequently, the wing beat transformations will be more complex. Similarly, there are few fluttering winged vehicles with autonomous takeoff and landing capabilities, and thus, the study of full-phase fluttering kinematics with bone transformations can likewise fill this gap. Meanwhile, the aerodynamic analysis based on kinematic data is also lacking. Real data do not support the existing aerodynamic simulation of bird size, and the motion parameters are mostly defined as simple transformations. The analysis of the aerodynamic performance of birds through real flight data is also a future research focus.

## 5. Conclusions

The current study comprehensively describes live pigeon free-flight wing kinematics. More 3D position trajectories at different locations on the fuselage and wing were obtained with higher accuracy than results from previous studies. Based on 3D data from anatomical points on the wing, we described the flapping process using five biologically relevant parameters that can be used to characterize avian aerodynamics. Additionally, a global optimization algorithm is proposed to compute the minimum Euclidean distance between a three-bar mechanism and the corresponding markers on the forearm of a real pigeon, which can be combined with cluster analysis to obtain the shoulder, elbow, and wrist angles during free-flight, which is rarely addressed in existing studies. We coupled the angles inside and outside the stroke plane by analyzing the correlation between the wing kinematics and the joint movement. This guides the configuration of the DOF of the bionic bird-inspired robot.

In future studies, unsteady aerodynamics can be evaluated through experimental and simulation studies of five-wing kinematics. In addition, DOF configuration is needed to design a vehicle comparable to the flight capabilities of birds. Other types of flight, such as take-off and landing, should also be analyzed. The current wing motion data can be used as a basis for future studies of more complex motions.

## Data Availability

All data that support the findings of this study are included within the article (and any Supplementary Files).

## Supplementary Materials

The following supporting information can be downloaded at: https://www.mdpi.com/2313-7673/9/9/555/s1. The Supplementary Materials.pdf contain detailed additions to the paper. Movie S1 shows the labeling in Pigeon. Movie S2 contains Pigeon in free flight and motion capture view. Data.zip contains the marker data from Pigeon.
