# Supplementary material for: Characterization of Wing Kinematics by Decoupling Joint Movement in the Pigeon
Source: Biomimetics (Basel). 2024 Sep 15;9(9):555. doi: 10.3390/biomimetics9090555 (PMC12068018; doi:10.3390/biomimetics9090555)
Supplement: Supplementary file 1 [file biomimetics-09-00555-s001.zip › Supplementary Materials.pdf]

**Supplementary Information**

**Supplementary Video S1: The marker points placement five pigeons and the labels in Motion capture system.**

**Supplementary Video S2: The GoPro camera perspective and motion capture view of five pigeon in free flight. The playback speed was set to 0.3 times.**

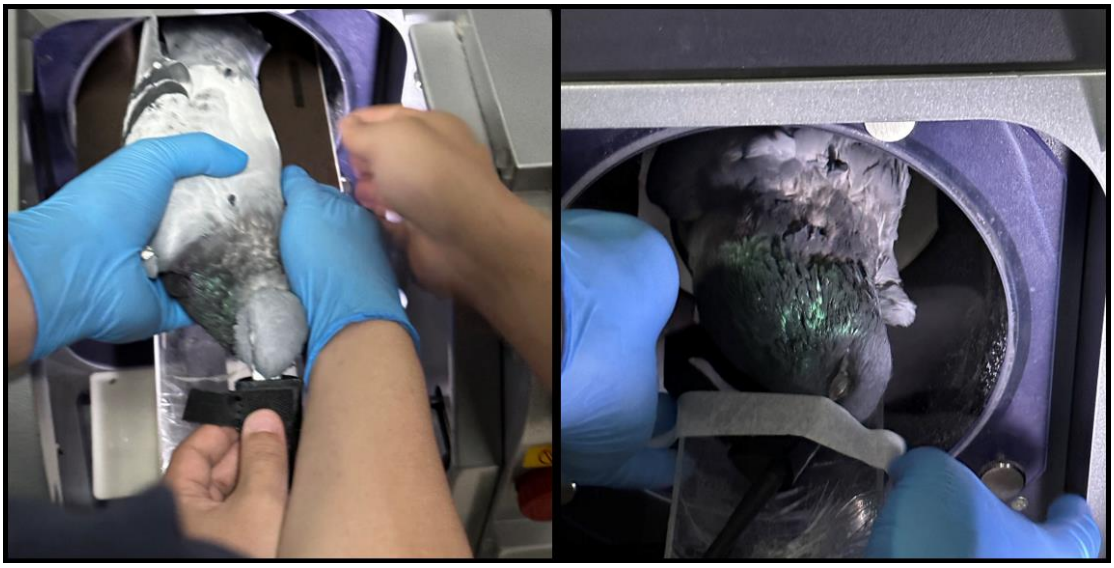

**Fig. S1.** Pigeons anaesthetized and placed into PE Quantum FX scanner for $CT$ scan.

**Table S1.** $\mu CT$ **results of forelimbs skeleton length for pigeons**

| Pigeon ID | Humerus (mm) | Radius and Ulna(mm) | Carpometacarpus (mm) |
|---|---|---|---|
| 4036 | 44.2 | 55.8 | 33.0 |
| 2417 | 43.3 | 56.9 | 33.7 |
| 2205 | 45.0 | 55.1 | 35.3 |
| 5018 | 46.3 | 58.3 | 36.5 |
| 2196 | 45.2 | 53.9 | 32.3 |

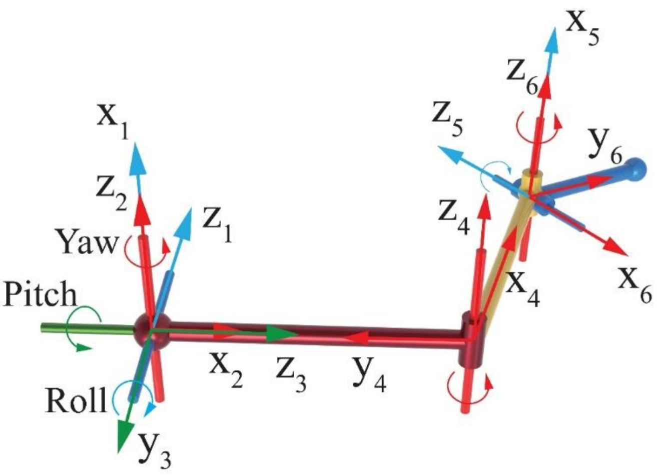


**Fig. S2.** Coordinate representation of the three-bar mechanism using the modified DH method. Pitch angles are depicted using a green coordinate system, Yaw is represented in red, and Roll is represented in blue. The red rod represents the arm, connecting the shoulder joint to the elbow joint. The yellow rods represent the forearm, connecting the elbow joint to the wrist joint. The blue rod represents the hand, connecting the wrist joint to the digit joint.

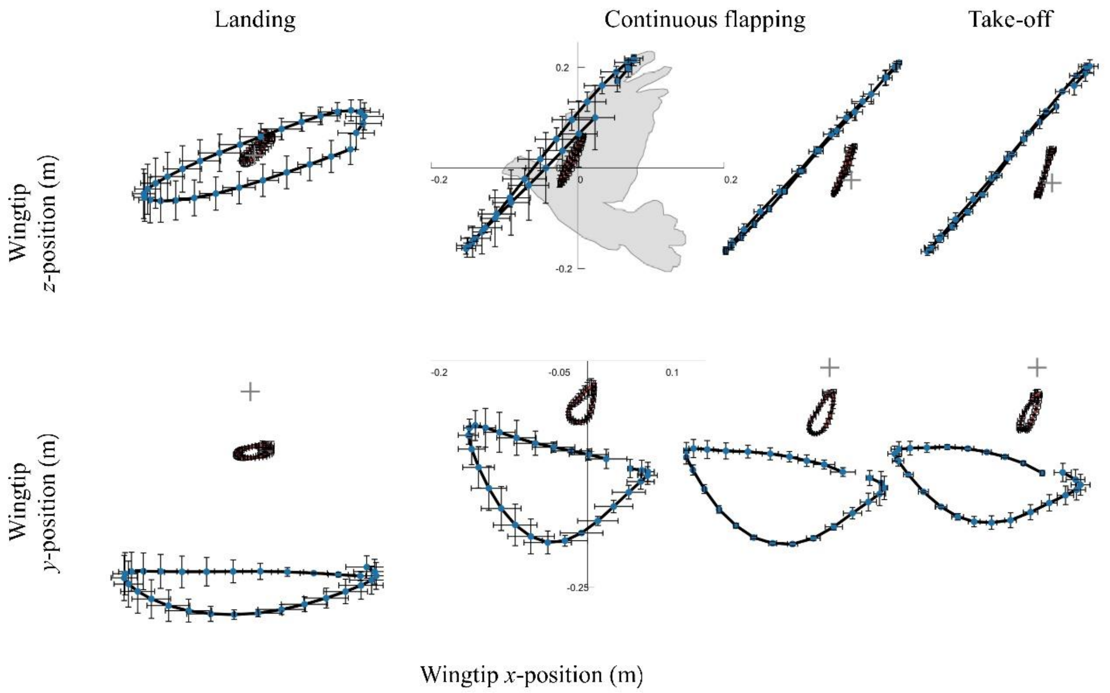


**Fig. S3.** Lateral and dorsal views illustrating the flight kinematics for each flight phase with pigeon ID 4036. The paths of P9 and WR are indicated by blue circles and orange circles, respectively. The cross hair is mean ± s.d. position from 24 flight sequences. '+' symbol denotes the middle position of LS and RS.

Overview of pigeon free flight behavior

The pigeon flight sequences in Movie 2 are from the GoPro camera and motion capture views, respectively. An example of the average trajectory sequence of the ninth primary and wrist in the body coordinate system projected onto the lateral and dorsal views is shown in Fig. S3. From right to left, the four processes that characterize the flight of a pigeon are: take-off, continuous flapping, gliding, and landing. The lateral view shows that from take-off to landing, the trajectory of ninth primary gradually transitions from a 'figure-of-eight' to an elliptical path, and the eccentricity of this ellipse gradually decreases. The trajectory of wrist transitions from a near line path to an elliptical path, and the wrist position is more caudal during downstroke than during upstroke. The trajectory illustrates the change in the stroke plane from a steep angle at takeoff to a gentle angle at landing. Dorsal observations showed that wingspan and wristspan were always maximal at mid-downstroke and minimal at mid-upstroke. The ninth primary was most caudal at the upstroke/downstroke transition. Simultaneously, the compressed appearance of the ninth primary path in the dorsal view during take-off is due to the steep stroke plane angle relative to the horizontal plane.

Compared to the other three flight phases, the continuous flight process is the most used in bird-inspired robots and therefore the most instructive. Hence, this phase was chosen for the wingbeat motion analysis in this paper. In addition, to eliminate the effect of the pigeon body orientation, we used the three angles defined earlier for selecting forward flight, i.e. $|\Theta| \leq 5$ deg, $|\Psi| \leq 5$ deg, $|\Phi| \leq 5$deg.

The number of wingbeats between perches for each pigeon ID was 21.14 $\pm$ 2.04. For each flight sequences, in addition to the first 5 wingbeats used for take-off, the next 2 wingbeats were analyzed. For each pigeon, several trials were carried out separately and 6 of them were taken. Taking into account the symmetry of the left and right wings, 24 wingbeats were collected from each pigeon ID, giving a total of 120 wingbeats.

**Table S2. Morphological parameters for pigeons**

| Pigeon ID | Mass (g) | Wing length (cm) | Wing area (cm^2) | Wing chord (cm) |
|---|---|---|---|---|
| 4036 | 400.4 | 61 | 621 | 10.18 |
| 2417 | 385.6 | 59 | 599 | 10.15 |
| 2205 | 419.5 | 58 | 613 | 10.57 |
| 5018 | 404.3 | 62 | 672 | 10.84 |
| 2196 | 393.2 | 64 | 655 | 10.23 |

**Table S3. Hyperparameters of optimization problem**

| Parameter Name | Description | Value |
|---|---|---|
| Max Iterations | Maximum number of iterations | 200 |
| Step Tolerance | Termination tolerance on | 2.1e-6 |
| Function Tolerance | Value for Convergence | 2.1e-6 |

**Aerodynamics metrics:**

To characterize the flight aerodynamics, three dimensionless parameters related to the wing morphology and movement were chosen: the Reynolds number ($R_e$), the Rossby number ($R_o$) and the advance ratio ($J$). These are the key parameters related to the Navier-Stokes equation.

$R_e$ determines the transition from laminar to turbulent flow in the wing boundary layer, which can be up to six orders of magnitude, ranging from close to 1 for the smallest hovering insect to about $10^6$ for a diving falcon, and is defined as:

$$R_e = \frac{U_f * \bar{c}}{v} ,$$

where $U_f$ is the forward velocity, $\bar{c}$ is the mean chord length, and $v$ is the kinematic viscosity (14.8*$10^6$ $m^2/s$). where the velocity is calculated using two-sided finite difference schemes:

$$u_i = \frac{(x_{i-2} - 8 * x_{i-1} + 8 * x_{i+1} - x_{i+2})}{(12 * \triangle t)} ,$$

where $x$ refer to position, and $\triangle t$ is the time (1/240 $s$).

However, because there is significant acceleration and deceleration during each wing stroke, $Re$ is not sufficient to characterize the flow around the flapping wing. $J$ is used to describe the effect produced by the reciprocating motion of the flapping wing and is described as:

$$J = \frac{U_f}{2 * A * f} ,$$

where $A$ is the flap amplitude and $f$ is the flap frequency. $J$ controls the time scale of vortex growth and shedding in the wake, so different vortex wake structures are formed at different values of $J$. Another dimensionless parameter is $R_o$, which is used to describe the ratio of the inertial force to the Coriolis force and is expressed as:

$$R_o = \sqrt{J^2 + 1} * \frac{R}{\bar{c}} ,$$

where $R$ is the wing length. $R_o$ controls the wing's ability to maintain a stable leading edge vortex (LEV), because it is inversely proportional to the centripetal Coriolis acceleration, inducing a steady flow of flapping wings.

**Table S4. Parameters that determine flapping wing aerodynamics**

| Pigeon ID | | 4036 | 2417 | 2205 | 5018 | 2196 |
|---|---|---|---|---|---|---|
| $U_f(ms^{-1})$ | mean | 6.09 | 6.17 | 6.68 | 6.42 | 5.05 |
| | s.d. | 0.10 | 0.04 | 0.35 | 0.23 | 1.33 |
| $R_e(*10^3)$ | mean | 4.19 | 4.23 | 4.77 | 4.69 | 3.49 |
| | s.d. | 0.07 | 0.02 | 0.25 | 0.17 | 0.92 |
| $J$ | mean | 0.75 | 0.88 | 0.84 | 0.83 | 0.72 |
| | s.d. | 0.01 | 0.36 | 0.05 | 0.04 | 0.36 |
| $R_O$ | mean | 7.48 | 7.86 | 7.17 | 7.41 | 7.77 |
| | s.d. | 0.04 | 1.40 | 0.19 | 0.15 | 0.79 |

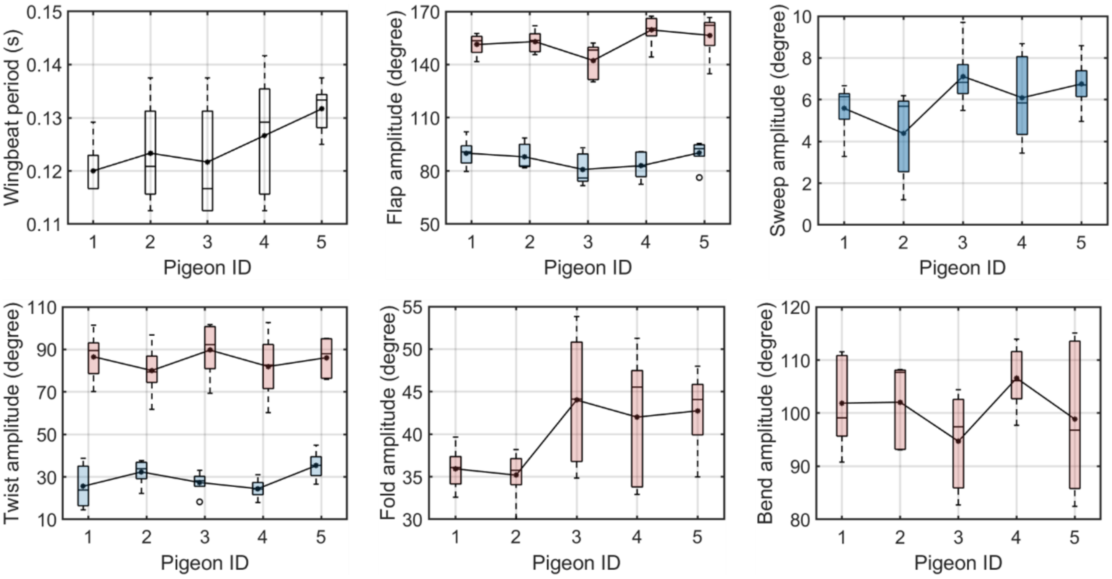


**Fig. S4.** Differential Testing of DoF Angle Changes Among Pigeons

**Systematic evaluation of joints angle configurations:**

The error chart in Fig. S5(a) shows that combinations with lower errors typically exhibited three or two DOFs in the shoulder joint, representing the shoulder joint as either a spherical or universal joint. The absence of the pitch angle had the smallest impact on error, suggesting that the model's error could be partially compensated by yaw and roll when the pitch angle was lacking. When only the yaw angle was absent, the model error escalated to 44.0 mm, and pitch and roll failed to effectively compensate for the absence of the yaw angle. Interestingly, the most substantial error arose when the motion of the shoulder joint was restricted to roll alone, indicating that the predominant motion of the forelimb was a flapping motion. However, as illustrated in Fig. S5(b), the angle of a specific shoulder joint consistently resulted in an error that was much greater compared with that when all three shoulder joint angles were present. Consequently, we determined the shoulder joint type to be spherical.

There were two configurations with the smallest errors among the combinations: the wrist joint using a spherical joint and a universal joint, and the shoulder joint as a spherical joint. Fig. S5(c) shows that the roll degree of the wrist joint had the most significant impact on the error, and the mean error with roll was 3.7 times smaller than that without roll angle. This result demonstrates that there was always a presence of roll angle in the configuration of the wrist joint angle.

Finally, when there was a consistent presence of roll angle in the wrist joint, the impact of pitch and yaw on the error was minimal. As shown in Fig. S5(d), when there was a pitch angle in the wrist, the error was 2.9mm, whereas without the pitch angle, it was 3.1mm. The marginal difference between the two values demonstrated that the influence of the pitch angle on the wrist joint was negligible. Taking into account the rigid linkage between feathers and bones, we ascertained that the wrist joint was a universal joint (with yaw and roll). Thus, the three joints can be represented as a spherical joint for the shoulder joint (3-DOFs), a pin joint for the elbow joint (1-DOF), and a universal joint for the wrist joint(2-DOFs).

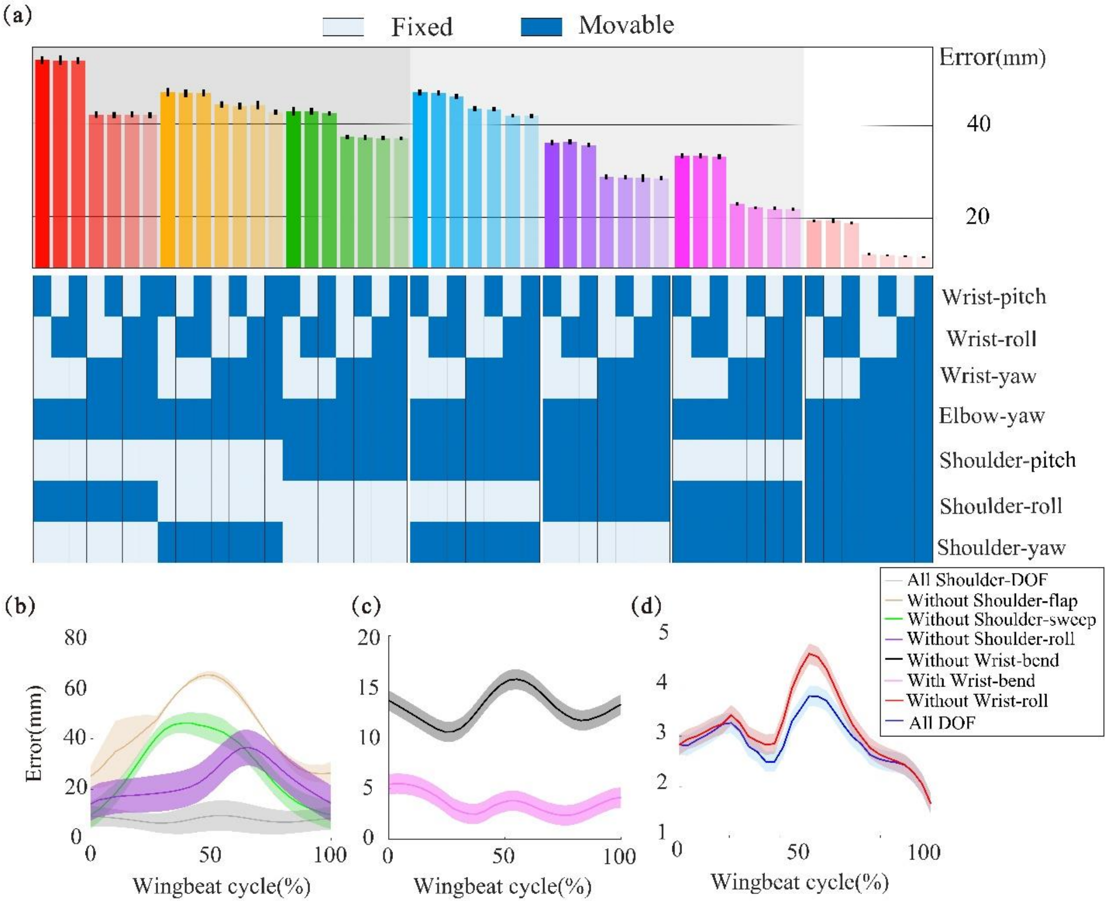


**Fig. S5.** Joint angle configuration and error comparison: (a) The above section indicates the error corresponding to each DOF combination. The shaded area on the left in gray represents the error corresponding to the shoulder joint as a pin joint, the middle section represents the error for the shoulder joint as a universal joint, and the white portion signifies the error for the shoulder joint as a spherical joint. The bottom section represents the arrangement and combination of joint angle. The blue grid indicates that the corresponding DOF is movable, and the white color indicates that it is fixed. (b) The

error comparison between the spherical joint and the universal joint for the shoulder joint. The mean error without shoulder roll angle is 44.0 mm, the mean error without shoulder yaw angle is 28.9 mm, the mean error without shoulder pitch is 23.14 mm and the mean error with spherical joint of shoulder is 8.2 mm. (c) The error comparison between the spherical joint and the universal joint for the wrist joint, the mean error with wrist roll is 3.4 mm, without wrist roll is 12.6 mm. (d) The error comparison between wrist with or without wrist pitch angle. The mean error with wrist pitch is 2.9 mm and without pitch is 3.1 mm.

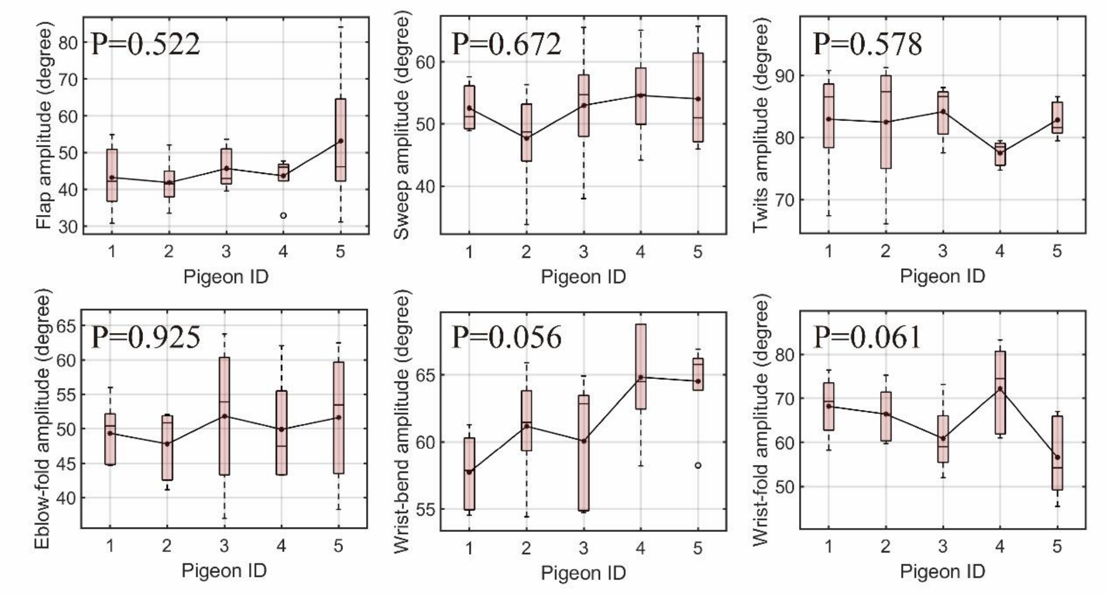


**Fig. S6.** Differential Testing of Joint Angle Changes Among Pigeons
